# A nested case-control study on the relationship between gut microbiota and non-alcoholic fatty liver disease

**DOI:** 10.3389/fendo.2026.1917815

**Published:** 2026-10-02

**Authors:** Xiangcheng Yang, Jingfeng Chen, Jianan Song, Haoshuang Liu, Lin Wang, Tiantian Li, Hang Yan, Xinxin Gao, Suying Ding

**Affiliations:** 1 Health Management Center, The First Affiliated Hospital of Zhengzhou University, Zhengzhou, Henan, China; 2 Department of Epidemiology, College of Public Health, Zhengzhou University, Zhengzhou, Henan, China; 3 Department of Occupational and Environmental Health, School of Public Health, Huazhong University of Science and Technology, Wuhan, Hubei, China

**Keywords:** gut microbiota, machine learning, metagenomic sequencing, microbial biomarkers, non-alcoholic fatty liver disease

## Abstract

**Background:**

The gut microbiota plays an important role in non-alcoholic fatty liver disease (NAFLD). This study aimed to explore gut microbiota-associated biomarkers for incident NAFLD and evaluate their predictive value, providing preliminary evidence for early risk identification.

**Methods:**

From an initial cohort of 1,772 participants, 404 were included in the final analysis, comprising 101 incident NAFLD cases and 303 age-matched controls (1:3). Gut microbiota composition and function were characterized by metagenomic sequencing. Microbial features were compared between participants who developed NAFLD (NAFLD -/+) and those who did not (NAFLD -/-). A CatBoost model integrating gut microbiota, functional pathways, and clinical indicators was constructed to predict NAFLD, with a random forest (RF) model on the same features for comparison. Progressively enriched feature sets assessed the incremental value of microbiota-related features beyond clinical indicators, and SHapley additive exPlanations (SHAP) values interpreted the model outputs.

**Results:**

The NAFLD (-/+) group had significantly lower species-level beneficial gut microbiota (*P* < 0.05), and the two groups differed markedly in gut microbiota metabolic enrichment. The CatBoost model achieved an area under the curve (AUC) of 0.762, indicating moderate predictive performance for incident NAFLD. We also identified significant intergroup differences in gut microbiota composition within the diagnostic model, including the key species *Coprobacillus unclassified* and the metabolic pathway PWY-5030.

**Conclusion:**

Gut microbiota alterations may serve as potential early biomarkers for incident NAFLD and contribute to its pathogenesis. The CatBoost model integrating multi-omics and clinical data shows moderate predictive ability, supporting the potential of gut microbiota-based approaches for non-invasive risk stratification.

## Introduction

Excessive hepatocyte fat accumulation is the hallmark of non-alcoholic fatty liver disease (NAFLD), a chronic liver illness that is unrelated to alcohol use or other recognized causes of liver damage. According to the 2023 multi-society Delphi consensus, NAFLD has been renamed MASLD; given their near-complete overlap, these terms are used interchangeably in this report (1). By 2030, it is expected to be among the most common reasons for liver transplants (2). NAFLD is now one of the main global public health concerns because it can have serious repercussions such liver cirrhosis and hepatocellular carcinoma, which have a significant negative impact on both national health and economic development (3). NAFLD is still not well known by the general public, although it impacts over 25% of the world’s population and placing a significant illness burden on human health (4). The main cause of this lack of awareness is that the majority of NAFLD patients do not exhibit any overt symptoms in the early stages of the illness and are typically discovered by chance during normal laboratory testing, imaging exams, or abdominal ultrasonography (5, 6). This frequently leads to an advanced diagnosis of illness, missing the chance for early care. However, despite their great diagnostic reliability, liver biopsy and imaging tests are not appropriate for widespread screening or monitoring. Theoretically, the progression of NAFLD can be stopped if proactive lifestyle changes are put in place and the condition is detected early (7). Therefore, it is urgent to develop an approach for identifying individuals at high risk of developing NAFLD or those with early-stage NAFLD.

Over the past decade, a large body of research has demonstrated that the gut microbiome is a key regulator of energy homeostasis and substrate metabolism (8–10). There are as many as 4,644 bacterial species in the human gastrointestinal tract, with a total of 171 million encoded genes (11). This implies that structural or functional abnormalities of the gut microbiome may exert an impact on the metabolism of the brain, adipose tissue, muscle and liver. Microbially derived compounds or metabolites, such as lipopolysaccharide (LPS), secondary bile acids, dimethylamine and trimethylamine, as well as fermentation products of carbohydrates and proteins, are closely associated with the intestinal host-microbiome metabolic axis and tightly linked to the development of metabolic diseases (12–15).

The significance of the gut microbiota in the development of NAFLD has become clearer in the realm of human cross-sectional investigations. Several studies have suggested that the risk of NAFLD progression may be linked to a higher ratio of Bacteroidetes to Firmicutes and a lower abundance of butyrate-producing Ruminococcaceae. Nonetheless, there is still inconsistency in the pertinent data (16–19). Furthermore, it is yet unknown if the dysbiosis seen in NAFLD patients is what accelerates the course of the disease or if NAFLD might cause notable taxonomic and functional changes in the gut microbiota. According to the possible causal relationship in the development of NAFLD, changes in the gut microbiota should occur before the disease is clinically diagnosed. This suggests that evaluating the gut microbiota in high-risk individuals may have significant predictive value for predicting when NAFLD will manifest.

This study constructed a machine learning (ML) model that integrates baseline gut microbial features, metabolic pathways, and basic clinical indicators to predict individuals’ NAFLD status over the next four years (remaining disease-free or developing the disease). These exploratory findings may help inform the development of preventive strategies for high-risk individuals undergoing physical examinations, offer a preliminary basis for the early identification of NAFLD risk, and provide initial clues for future research on disease intervention and health management.

## Materials and methods

### Study population

This study enrolled individuals who underwent routine health check-ups at the First Affiliated Hospital of Zhengzhou University from January 2018 to February 2019. Inclusion criteria were as follows: 1) Healthy check-up participants aged 18 years and above; 2) No fatty liver disease at the initial check-up; 3) Provision of informed consent. Exclusion criteria were as follows: 1) A history of alcohol consumption, or average weekly alcohol intake equivalent to ≤210 g of ethanol for males and ≤140 g for females; 2) Comorbidities including malignant tumors, autoimmune liver disease, Wilson’s disease, liver cirrhosis, hepatocellular carcinoma, viral hepatitis, drug-induced fatty liver disease and other liver disorders that can cause fatty liver, as well as abnormal renal function; 3) Incomplete check-up data; 4) A history of gastrointestinal diseases (e.g., inflammatory bowel disease, irritable bowel syndrome) or use of antacids, gastric mucosal protectants, antibiotics or probiotics within the previous 3 months; 5) Pregnant or lactating women.

Initially, we registered 1,772 participants. After applying the exclusion criteria and follow-up assessment, we identified 101 participants who were free of NAFLD at baseline but developed NAFLD during follow-up as cases. We selected 303 controls from participants who remained free of NAFLD throughout follow-up using an age-based 1:3 control selection strategy. Finally, we included 404 participants in the final analytic sample, including 184 men and 220 women. **Appendix Figure S1** shows the participant selection process.

### Ethical statement

The Ethics Review Committee of Zhengzhou University’s First Affiliated Hospital granted approval for this study (Approval No. 2018-KY-90), and it was carried out in compliance with the Declaration of Helsinki’s ethical guidelines. After thoroughly comprehending the study’s goals and substance, each participant completed the informed consent form. Researchers made sure that participants understood the study completely, that their involvement was completely voluntary, and that their medical care would not be affected if they choose to leave the study at any point.

Personal information was strictly confidential and only accessible to the principal researchers. Identifiable information was de-identified prior to data analysis to protect privacy. Only aggregated data were presented in study reports and academic papers, with no individual identities involved. All researchers received ethical training, adhered to privacy and data protection standards, and were under the supervision of the Ethics Committee.

### Data collection

Data on demographics, medical history, and lifestyle factors, such as smoking, drinking, eating, and exercising, were gathered using a semi-structured questionnaire survey based on interviews. Baseline screening was performed by trained medical personnel who used standardized self-administered electronic questionnaires to collect information on gender, age, height, weight, and waist circumference (WC). Blood pressure was measured with an automated sphygmomanometer in a quiet environment, including diastolic blood pressure (DBP) and systolic blood pressure (SBP). For biochemical index detection, a Mindray BC-6800Plus five-part differential hematology analyzer (Mindray, China) was used to measure complete blood count (CBC) indices, including white blood cell count (WBC) and red blood cell count (RBC). In addition, a Roche Cobas-8000 fully automated biochemistry analyzer (Roche, Germany) was employed to detect biochemical parameters, such as blood lipids (TC, TG, HDL-C, and LDL-C), fasting blood glucose (FBG), γ-glutamyl transferase (GGT), alkaline phosphatase (ALP), total protein (TP), albumin (ALB), total bilirubin (TBIL), blood urea nitrogen (BUN), creatinine (CREA), and serum uric acid (SUA). Fecal samples were immediately frozen in liquid nitrogen after collection and stored in an ultra-low temperature refrigerator at -80°C. Upon completion of sample collection, laboratory staff were notified promptly for testing, with the entire sampling process controlled to be completed within 1–2 hours. According to the diagnostic criteria for NAFLD formulated by the Fatty Liver Disease Professional Committee of the Hepatology Society of the Chinese Medical Doctor Association in 2018, a diagnosis of diffuse fatty liver disease can be made if at least two of the following three criteria are met via abdominal ultrasound examination (20): 1) Diffuse hyperechogenicity is observed in the near field of the liver (i.e., “bright liver”), with hepatic echogenicity higher than that of the kidney; 2) The intrahepatic ductal structures cannot be clearly visualized; 3) Gradual attenuation of echogenicity is noted in the far field of the liver.

### Diagnostic criteria for NAFLD

Abdominal ultrasound was used in this study to confirm the imaging evidence of NAFLD (20), and the diagnostic criteria for NAFLD required the fulfillment of the following three basic conditions:1) No history of alcohol consumption, or an average weekly alcohol intake equivalent to ≤210 g of ethanol for males and ≤140 g for females; 2) Exclusion of other liver diseases that can cause fatty liver, including autoimmune liver disease, Wilson’s disease, viral hepatitis, and drug-induced fatty liver disease; 3) Evidence of hepatic steatosis confirmed by imaging or histological examinations.

### Metagenomic sequencing of gut microbiota

The details of instruments and reagents required for the fecal DNA extraction experiment, including their manufacturers, are as follows: Thermomixer (Eppendorf AG, Germany); low-temperature centrifuge (Eppendorf AG, Germany); vortex mixer (Haimen Kylin-Bell Instrument Manufacturing Co., Ltd., China); anhydrous ethanol (Guangdong Xilong Chemical Co., Ltd., China); fecal DNA extraction kit (Guangzhou Meiji Biotechnology Co., Ltd., China). The specific experimental procedures for DNA extraction were performed in accordance with the manufacturer’s instructions. Metagenomic libraries were constructed using fecal DNA, with the specific protocol as follows: DNA sample quality control (detection of concentration, integrity and purity); DNA fragment selection (targeting sample bands of approximately 200–400 bp); reaction system establishment (end repair, adapter ligation, polymerase chain reaction (PCR), magnetic bead purification and circularization reaction); and sequencing (MGI-SEQ-2000 sequencing platform (BGI, China)). Raw sequencing reads were filtered to remove the following sequences: those containing sequencing adapters, those with low-quality bases (base quality ≤5) accounting for more than 50% of the total sequence length, and those with unknown bases (N) exceeding 10%.

In the metagenomic analysis, MetaPhlAn2 software (21) was used for taxonomic annotation of the sequencing libraries, aiming to obtain the standardized relative abundance values of each species across different taxonomic ranks. The analysis process first involved aligning metagenomic sequences against a precomputed marker gene catalog via BLAST nucleotide search, which allowed the characterization of species diversity for each metagenomic sample. Considering that different subclades possess unique marker genes, the classifier performed normalization by dividing the number of reads mapped to each phylogenetic clade by the indexed nucleotide length and then calculated the relative abundance of each taxonomic unit. Subsequently, microbial abundance was estimated through standardization against the average genome size of each phylogenetic clade, thereby generating a comprehensive taxonomic profile of the gut microbiota encompassing bacteria, archaea, eukaryotes, and viruses. The National Center for Biotechnology Information (NCBI) database and HMP Unified Metabolic Analysis Network 2 (HUMAnN2) were employed to annotate the non-redundant gene sets and functional genes, enabling the generation of a comprehensive overview of metabolic pathways (22, 23).

### Statistical analysis

We used SPSS 26.0 to analyze demographic characteristics and laboratory test results. We summarized categorical variables as frequencies and percentages and compared them between groups using the chi-square test or Fisher’s exact test, as appropriate. We assessed the normality of continuous variables using the Kolmogorov-Smirnov test and expressed them as mean ± standard deviation or median with interquartile range, as appropriate. We compared continuous variables between groups using Student’s t-test or the Mann-Whitney U test according to data distribution and homogeneity of variance.

To analyze the gut microbiota community structure, the Bray–Curtis distances between samples were calculated using the “vegan” package in R software (v4.5.3), and the total variance of these distances was partitioned. Furthermore, to more intuitively understand the relationships between samples and environmental factors, redundancy analysis (RDA) was performed, and samples together with environmental factors were visualized on a two-dimensional ordination plot using the “ggplot2” and “ggrepel” packages. We compared α-diversity indices, including Shannon, Observed species, Simpson, and Gini indices, between the two groups using the Wilcoxon rank-sum test. We analyzed β-diversity using the Adonis function in the vegan package and compared intergroup differences based on Bray-Curtis distance, Jensen-Shannon divergence, Pearson coefficient distance, and Spearman coefficient distance. Principal coordinate analysis (PCoA)was performed on species-level relative abundances to visualize β-diversity between the NAFLD(−/+) and NAFLD(−/−) groups across five distance metrics. PCoA was conducted using dudi.pco in the R package ade4 (nf = 5), with the quasieuclid correction applied to non-Euclidean distances. The variance contribution of PC1 and PC2 was derived from eigenvalues. Between-group differences were tested using the Wilcoxon rank-sum test on PC1 for each distance metric. We set statistical significance at *P* < 0.05.

Prior to differential analysis, taxa and metabolic pathways with a prevalence of less than 10% were removed from the dataset to reduce false-positive results. The linear discriminant analysis effect size analysis (LEfSe) method was employed to identify statistically significant biomarkers between the two groups. This method consists of the following main steps:1)The Kruskal–Wallis rank-sum test was used to screen for taxa or metabolic pathways showing significant differences among groups;2)The Wilcoxon rank-sum test was applied to compare the two groups in a pairwise manner to further confirm the differences;3)Finally, the LDA score was used to assess the biological relevance or effect size of the significantly different taxa or metabolic pathways;4)Biomarkers with significant differences were defined as those with high LDA scores (e.g., LDA score > 2 or 3). In addition, Pearson correlation analysis was used to evaluate correlations. The significance level for differential comparisons and correlation analyses was set at *P* < 0.05.

This study applied Least absolute shrinkage and selection operator (LASSO) regression to screen candidate predictors associated with NAFLD and constructed a CatBoost model using R software. The dataset was randomly divided into a training set and a test set at a ratio of 8:2. Hyperparameter tuning relied on 5-fold cross-validation repeated five times within the training set, and the optimal classification threshold, determined by maximizing the Youden index, was applied unchanged to the test set. A random forest (RF) model trained on the same feature set served as a benchmark to justify the selection of CatBoost. To assess the incremental predictive value of gut microbiota and its metabolic pathway, three CatBoost models incorporated progressively enriched feature sets: (1) the nine traditional indicators retained by LASSO; (2) these indicators plus *Coprobacillus unclassified*; and (3) these indicators plus both *Coprobacillus unclassified* and the metabolic pathway PWY-5030. Model performance was evaluated using the area under the curve (AUC) with 95% confidence intervals estimated by the DeLong method, together with accuracy, sensitivity, specificity, recall, and F1 score. SHapley Additive exPlanations (SHAP) values quantified the contribution of each feature to the final model. Sex distribution was compared between groups and considered when interpreting the findings. **Figure 1** shows the overall workflow of the study.

**Figure 1:**
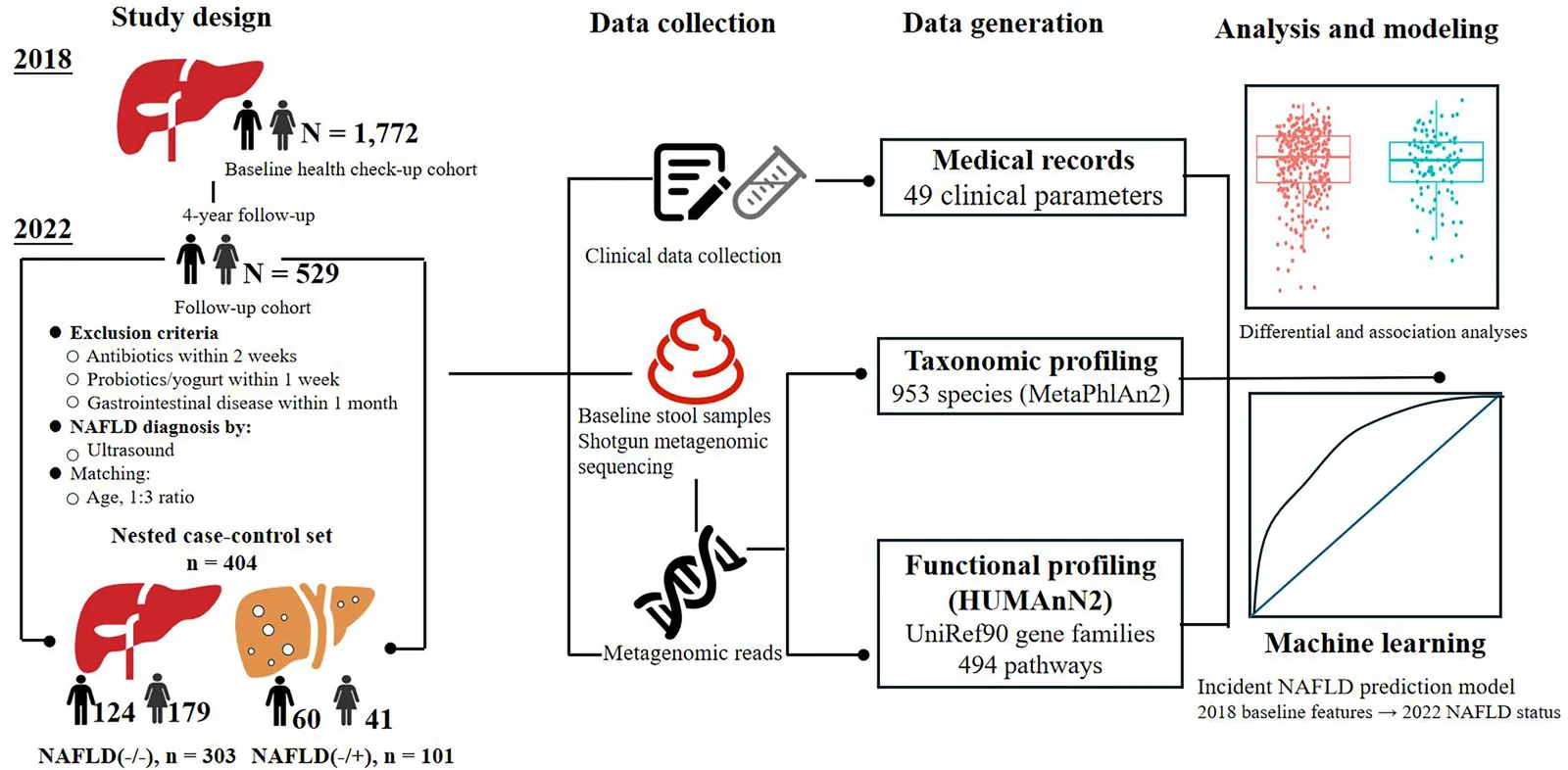
Study flowchart.

## Result

### Baseline characteristics of subjects

A total of 404 subjects were included in this study, among whom 101 were in the NAFLD (-/+) group (60 males, 59.41%; 41 females, 40.59%) with a mean age of 46.58 ± 8.46 years, and 303 were in the NAFLD (-/-) group (124 males, 40.92%; 179 females, 59.08%) with a mean age of 45.81 ± 8.25 years. Systolic blood pressure (SBP) (123.21 ± 12.86 mmHg vs. 118.62 ± 10.31 mmHg) and alanine aminotransferase (ALT) (22.47 ± 12.73 U/L vs. 18.20 ± 9.31 U/L) in the NAFLD (-/+) group were significantly higher than those in the control group (all *P* < 0.05). Demographic data between the two groups were compared using the chi-square test. The results showed that the proportion of subjects with regular physical activity in the NAFLD (-/-) group (61.06% vs. 46.53%) was significantly higher than that in the NAFLD (-/+) group (*P* < 0.05), while no significant differences were observed in other demographic and laboratory indicators between the two groups (*P*>0.05). Comparisons of the baseline characteristics and laboratory indicators of the study subjects are presented in **Table 1**.

**Table 1:** Comparisons of the baseline characteristics and laboratory indicators of the study subjects.

	NAFLD(-/+)(n=101)	NAFLD(-/-)(n=303)	t/W/*X*^2^	*P* value
Age^a^	46.58±8.46	45.81±8.25	-0.817	0.415
SBP(mmHg)^a^	123.21±12.86	118.62±10.31	4531.500	0.024
DBP(mmHg)^a^	75.32±9.83	73.64±11.08	1.814	0.180
BMI(kg/m^2^)^a^	24.05±2.44	23.99±3.05	2.452	0.119
Height(cm)^a^	167.74±7.80	167.82±8.27	0.311	0.578
Weight(kg)^a^	67.96±9.41	65.76±11.50	3.382	0.068
WC(cm)^b^	83.50 (75.00, 89.00)	83.00 (78.00, 88.00)	3920.500	0.682
Sex^c^				
Male	60(59.41)	124(40.92)	0.001	0.999
Female	41(40.59)	179(59.08)		
Regular diet^c^				
No	10(9.90)	36(11.88)	0.294	0.587
Yes	91(90.10)	267(88.12)		
Dietary habit				
Mixed	86(85.15)	251(82.84)	0.345	0.841
Meat	3(2.97)	9(2.97)		
Vegetarian	12(11.88)	43(14.19)		
Whole Grain^c^				
No	19(18.81)	70(23.10)	0.812	0.368
Yes	82(81.19)	233(76.90)		
Drinking^c^				
No	63(62.38)	147(48.51)	1.531	0.216
Yes	38(37.62)	156(51.49)		
Smoking^c^				
No	70(69.31)	251(82.84)	1.719	0.190
Yes	31(30.69)	52(17.16)		
Exercise^c^				
No	54(53.47)	118(38.94)	16.939	0.001
Yes	47(46.53)	183(61.06)		
ALP(U/L)^a^	68.23±17.77	66.13±18.73	-0.986	0.325
AST(U/L)^a^	20.50±6.69	19.55±5.79	-1.373	0.171
ALT(U/L)^a^	22.47±12.73	18.20±9.31	-3.103	0.002
GGT(U/L)^a^	26.52±43.96	18.24±11.82	-1.872	0.064
ALB(g/L)^a^	48.39±5.72	47.91±4.05	-0.909	0.364
TG(mmol/L)^b^	1.06 (0.79, 1.72)	1.30 (0.97, 1.82)	2022.000	0.054
TBIL(μmlo/L)^a^	11.81±5.00	11.50±5.22	0.395	0.856
TP(g/L)^a^	75.24±8.80	74.38±6.10	-1.078	0.282
ALB(g/L)^a^	48.39±5.72	47.91±4.05	-0.909	0.364
WBC(×10^9^/L)^a^	6.21±1.81	5.58±1.38	-3.242	0.067
RBC(×10^9^/L)^a^	4.73±0.65	4.62±0.52	-1.733	0.084
PLT(×10^9^/L)^a^	236.11±63.12	227.47±54.17	-1.232	0.220
BUN(mmol/L)^a^	4.48±0.98	4.36±1.08	-0.991	0.322
UA(μmol/L)^a^	310.10±75.26	309.43±64.12	0.064	0.332
CREA(mmol/L)^a^	71.54±15.06	71.30±13.15	0.113	0.080
TC(mmol/L)^a^	4.64±0.83	4.58±0.92	-0.467	0.253
TG(mmol/L)^b^	1.06 (0.79, 1.72)	1.30 (0.97, 1.82)	2022.000	0.054
HDL-C(mmol/L)^a^	1.45±0.31	1.42±0.38	0.400	0.223
LDL-C(mmol/L)^a^	2.77±0.77	2.82±0.69	-0.395	0.237
FBG(mmol/L)^a^	5.13±0.57	5.06±0.59	0.818	0.935
TyG^a^	6.92±0.26	6.92±0.24	0.659	0.418

WC: waist circumference; ALP: alkaline phosphatase; AST: aspartate aminotransferase; ALT: alanine aminotransferase; GGT: gamma-glutamyltransferase; TBIL: total bilirubin; TP: total protein; ALB: albumin; WBC: white blood cell count; RBC: red blood cell count; PLT: platelet count; BUN: blood urea nitrogen; UA: uric acid; CREA: creatinine; TC: total cholesterol; TG: triglyceride; HDL-C: high-density lipoprotein cholesterol; LDL-C: low-density lipoprotein cholesterol; FBG: fasting blood glucose; TyG: triglyceride-glucose index. .

a: Continuous variables are presented as mean (standard deviation), and intergroup differences were tested using the Student’s t-test.

b: Non-normally distributed continuous variables are presented as median (interquartile range), and intergroup differences were tested using the Mann-Whitney U test.

c: Categorical variables are presented as n (%), and intergroup differences were tested using the chi-square test.

### Relationships between the gut microbiota and environmental factors

This study performed RDA to explore the associations between gut microbiota and baseline characteristics of participants (**Appendix Figure S2**). From the perspective of environmental factors and microbial community distribution, the arrow representing age was the longest, indicating the strongest correlation with microbial community and sample distribution; the arrow representing the TyG index was the shortest, indicating the weakest correlation. The angles between the arrow of NAFLD and those of gender, smoking, alcohol consumption, body weight, WC, SBP, and DBP were acute angles, suggesting a positive correlation between NAFLD and the aforementioned environmental factors. In contrast, the angles between the arrow of NAFLD and those of physical activity, age, and dietary habits were obtuse angles, indicating a negative correlation between NAFLD and these environmental factors.

### Analysis of microbial diversity differences at the species level

To assess differences in species-level gut microbiota diversity associated with NAFLD, we first conducted α-diversity analysis. The results showed that no statistically significant differences were observed in the species-level diversity of the two groups when compared using the Shannon, Obs, Simpson, and Gini indices (Wilcoxon rank-sum test, *P*>0.05, **Appendix Figure S3**).

Based on the species-level composition of the gut microbiota, this study performed PCoA to investigate differences in the overall microbial composition (i.e., *β*-diversity) between the NAFLD (-/+) and NAFLD (-/-) groups (**Appendix Figure S4**). To ensure the robustness of the results, four distinct sample distance metrics were employed: Bray-Curtis Distance (**Appendix Figure S4A**), Jensen-Shannon Divergence (**Appendix Figure S4B**), Pearson-Coefficient Distance (**Appendix Figure S4C**), and Spearman-Coefficient Distance (**Appendix Figure S4D**). Results of Adonis analysis indicated that there were no statistically significant differences between the two groups across all four-distance metrics (*P*>0.05), and no significant differences in the community composition of the two groups were observed (*P*>0.05). A comprehensive analysis of four kinds of distance metrics revealed that there was no statistically significant difference in the *β*-diversity of the gut microbiota between the NAFLD (-/+) and NAFLD (-/-) groups (*P*>0.05).

### Analysis of gut microbiota structural differences between the NAFLD (-/+) and NAFLD (-/-) groups

A total of 953 species were taxonomically identified at the species level from all samples via sequencing. Based on species-level microbial composition, we performed LEfSe analysis to identify microbial taxa associated with NAFLD. The results revealed 44 species with statistically significant differences between the NAFLD (-/+) and NAFLD (-/-) groups (*P* < 0.05). Among these, 8 species were more abundant in the NAFLD (-/-) group than in the NAFLD (-/+) group, including *Bacteroides fragilis*, *Ruminococcus gnavus*, *Bacteroides dorei*, *Bacteroides caccae*, *Haemophilus parainfluenzae*, *Streptococcus salivarius*, *Parabacteroides johnsonii* and *Coprobacillus unclassified* (with LDA scores of 3.671, 3.520, 3.260, 3.233, 3.022, 2.867, 2.376 and 2.360, respectively). In contrast, 36 species were more abundant in the NAFLD (-/+) group than in the NAFLD (-/-) group, including *Bilophila wadsworthia*, *Eubacterium ramulus* and *Oxalobacter formigenes* (with LDA scores of 2.020, 2.041 and 2.242, respectively). **Figure 2** shows the species with significant intergroup differences at the species level.

**Figure 2:**
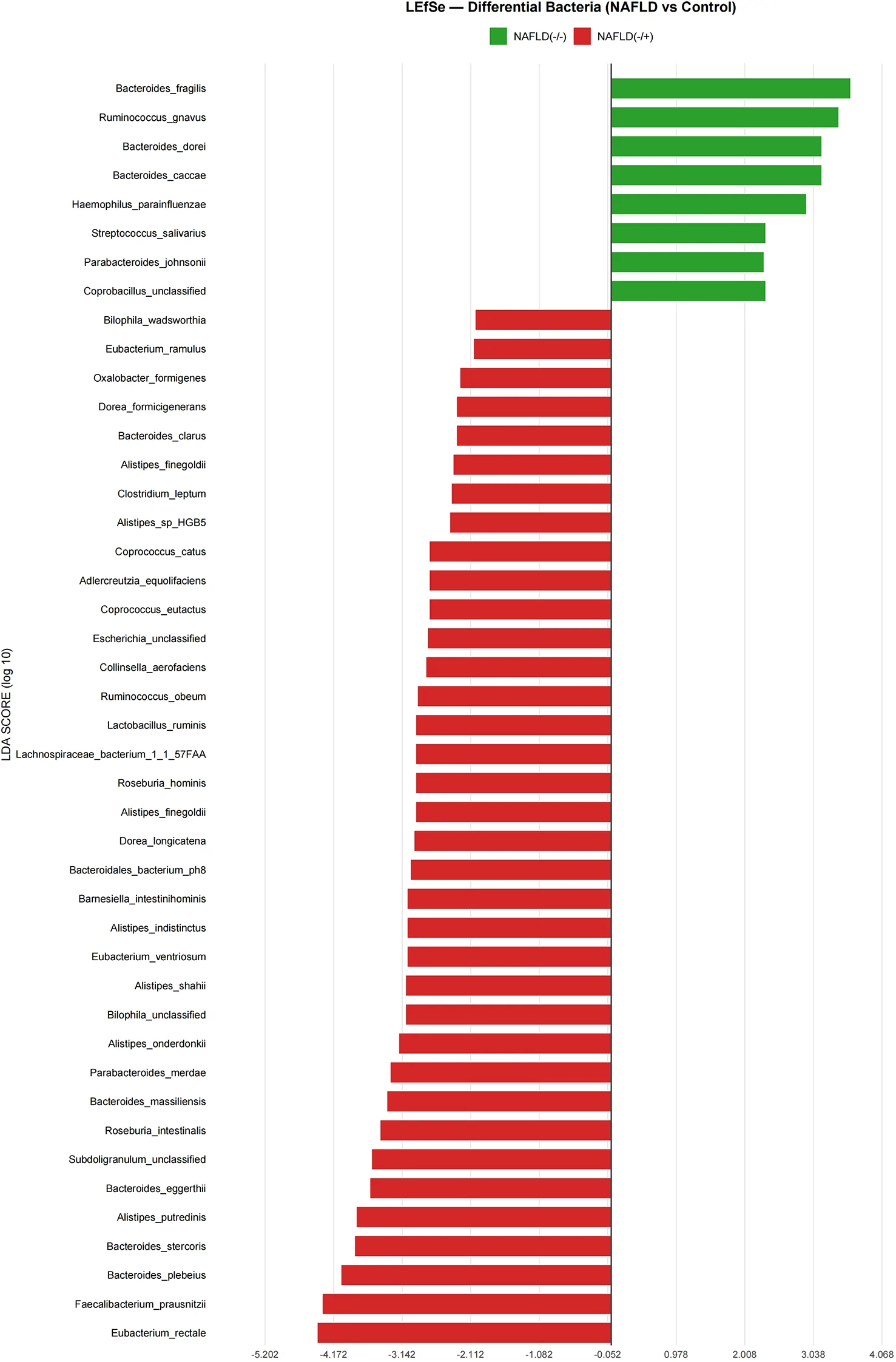
Differences in Gut Microbiota Abundance Between the NAFLD (-/+) and NAFLD (-/-) Groups.

### Correlations between the gut microbiota and clinical indicators

At the species level, Spearman’s correlation analysis was performed to assess associations between clinical indicators and gut microbial abundance, and a heatmap was generated to visualize these correlations. The results showed that *Ruminococcus obeum* was significantly negatively correlated with ALP and TG; *Coprococcus catus* was significantly positively correlated with CREA; and *Alistipes shahii* was significantly negatively correlated with FBG, TC, TG, DBP and BMI (**Figure 3**).

**Figure 3:**
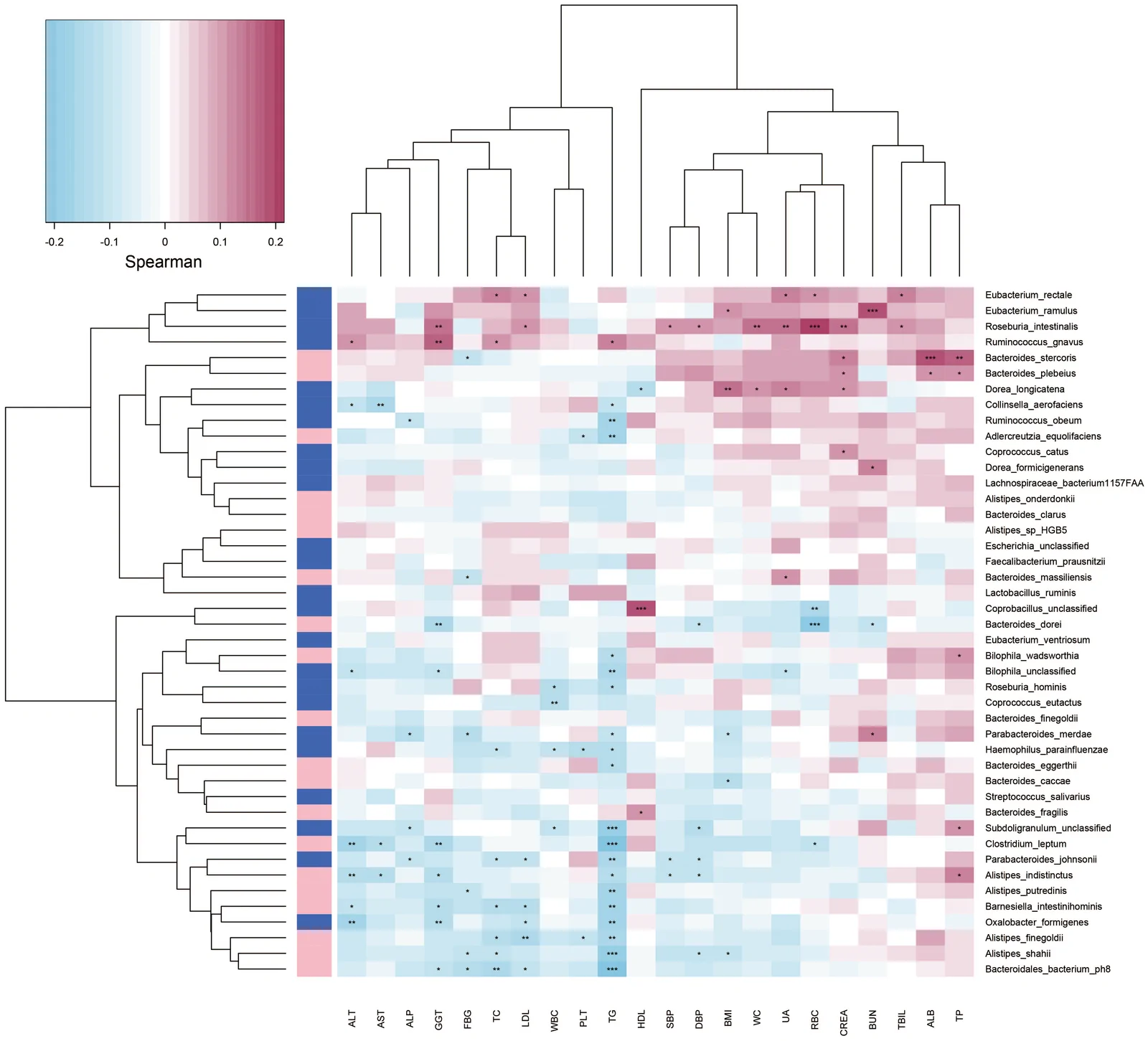
Correlations Between the Gut Microbiota and Clinical Indicators.

### Analysis of gut microbiota metabolic pathway differences between the NAFLD (-/+) and NAFLD (-/-) groups

This study simultaneously used the MetaCyc and KEGG metabolic pathway databases to analyze the enriched metabolic pathways of the gut microbiota in the NAFLD (-/+) and NAFLD (-/-) groups. We also performed LEfSe analysis, which identified 60 metabolic pathways with significant differences between the two groups (LDA>2, *P* < 0.05, **Figure 4**). Among these differential pathways, 21 metabolic pathways with significantly higher relative abundance in the NAFLD (-/-) group than in the NAFLD (-/+) group included PWY-6700, PWY4FS-7 and PWY4FS-8 (with LDA scores of 3.160, 2.751 and 2.750, respectively). In contrast, 39 metabolic pathways showed higher relative abundance in the NAFLD (-/+) group compared with the NAFLD (-/-) group, including SALVADEHYPOX-PWY, PWY-5030 and PWY-5345 (with LDA scores of 2.138, 2.152 and 2.220, respectively). **Figure 4** shows the microbial metabolic pathways with significant intergroup differences at the species level, while **Appendix Table S1** presents the relative abundances of all microbial metabolic pathways with significant differences. Information on the functional classification and the names of metabolic pathways are shown in **Appendix Tables S2**, **S3**. According to the statistical results, the gut microbiota in the NAFLD (-/+) group was enriched in lipid synthesis, carbohydrate metabolism (glycolysis), amino acid synthesis, amino acid activation, energy conversion, nucleotide synthesis, macromolecule catabolism and macromolecule anabolism.

**Figure 4:**
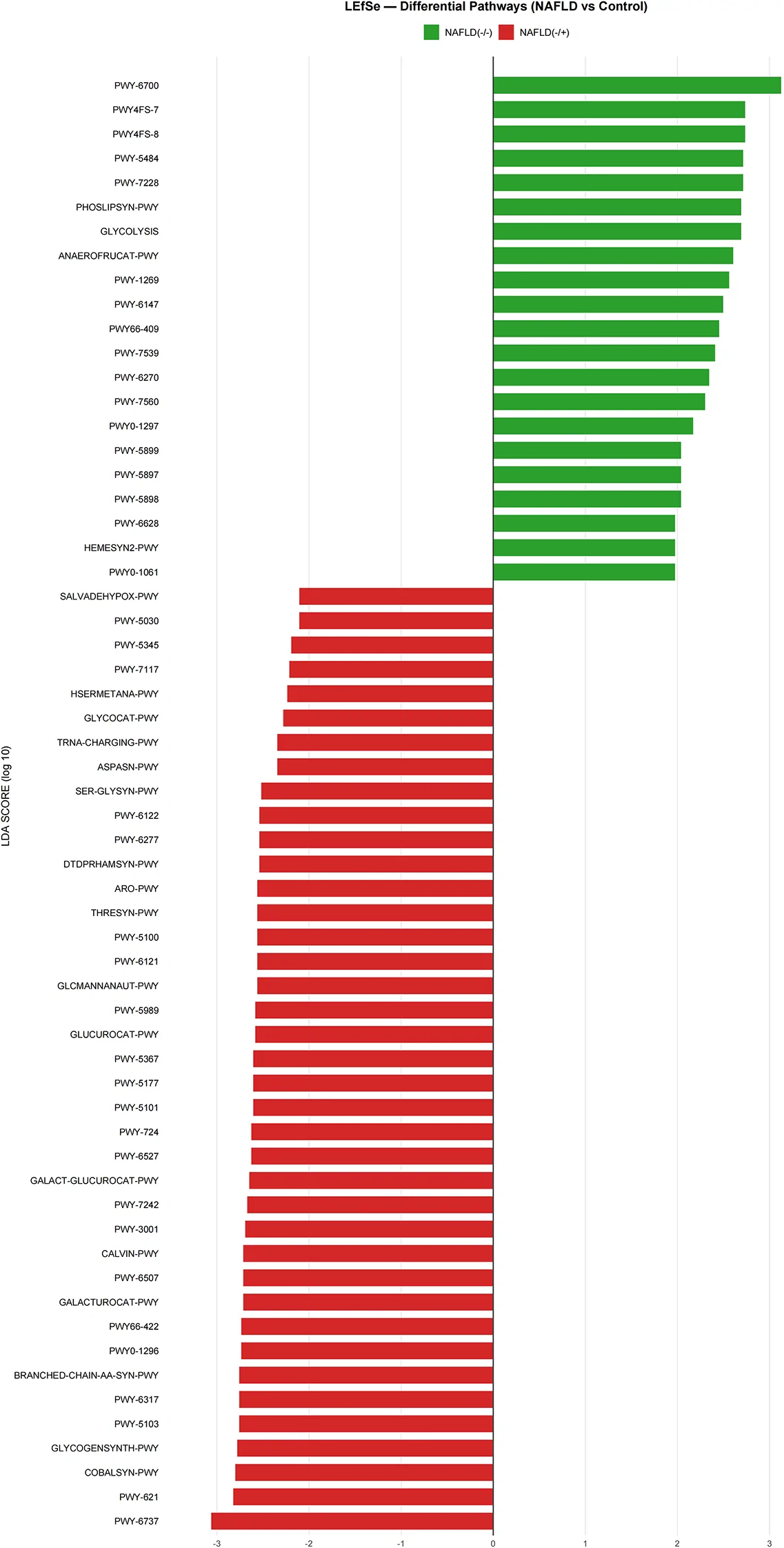
Differences in gut microbiota metabolic pathways between the NAFLD (-/+) and NAFLD (-/-) groups.

### Correlations between gut microbiota metabolic pathways and clinical indicators

The present study employed Pearson’s correlation analysis to investigate the associations between the abundance of gut microbiota metabolic pathways and clinical markers. According to the results, PWY-1269 had a significant positive correlation with UA and TG, PWY-5030 had a significant positive correlation with TBIL and a significant negative correlation with FBG, TG, and BMI, and PWY-5367 had a significant positive correlation with ALB, RBC, CREA, UA, TBIL, and WC (**Appendix Figure S5**).

### Lasso-based feature selection

The results of cross-validation were plotted to generate **Appendix Figure S6**. The upper abscissa represents the number of variables with non-zero coefficients in the current model, the lower abscissa is log(λ), and the ordinate is the deviance. The red dots in the figure indicate the target parameter corresponding to each λ value. The figure illustrates how deviance changes with different values of λ. The first vertical line from the left corresponds to lambda.min, which is the optimal λ value at the minimum deviance of the model; the second vertical line corresponds to lambda.1se, referring to the λ value of the simplest model obtained within one standard error of lambda.min. In this study, lambda.min was selected as the threshold for variable selection, and the calculated lambda.min value was 0.02775469. Ultimately, the number of variables was reduced to 9: WC, BMI, WBC, GGT, TP, TC, smoking, drinking, exercise.

### Construction and evaluation of the RF prediction model

To evaluate whether the selected core microbial species, gut microbiota metabolic pathways, and clinical indicators could effectively predict NAFLD status, a CatBoost prediction model was constructed based on these features and internal validation was performed. To justify the selection of CatBoost, a RF model was additionally trained on the same feature set and its predictive performance was compared with that of the CatBoost model in both the training and test sets.

The 11 features incorporated into the final model comprised the nine traditional clinical indicators retained by LASSO regression (WC, BMI, WBC, GGT, TP, TC, smoking, drinking, and exercise), together with the gut microbial taxon *Coprobacillus unclassified* and the metabolic pathway PWY-5030. *Coprobacillus unclassified* was additionally incorporated because it showed the strongest association with NAFLD among the candidate microbial species in the preliminary screening and is implicated in short-chain fatty acid metabolism, which has been linked to hepatic steatosis. PWY-5030 was further added as it ranked highest among the candidate metabolic pathways and is functionally related to amino acid metabolism relevant to NAFLD pathogenesis. The study population was randomly divided into a training set and a test set at a ratio of 8:2 (training set: 324 cases; test set: 80 cases). The training set was used for model construction, while the test set was used for model validation and evaluation.

The CatBoost model demonstrated favorable and stable predictive performance for NAFLD. In the independent test set, the model achieved an AUC of 0.762 (95% CI:0.647-0.878), with an accuracy of 0.725, sensitivity of 0.700, specificity of 0.733, recall of 0.700, and F1 score of 0.560 (**Figure 5A**). Compared with the RF benchmark trained on the same feature set (test set: AUC = 0.698 (95% CI:0.563-0.0.832), accuracy = 0.463, sensitivity = 0.900, specificity = 0.317, F1 score = 0.456), the CatBoost model yielded a higher overall accuracy, AUC, specificity, and F1 score, with a more balanced sensitivity–specificity trade-off, supporting its superior predictive ability for the present task (**Figure 5B**).

**Figure 5:**
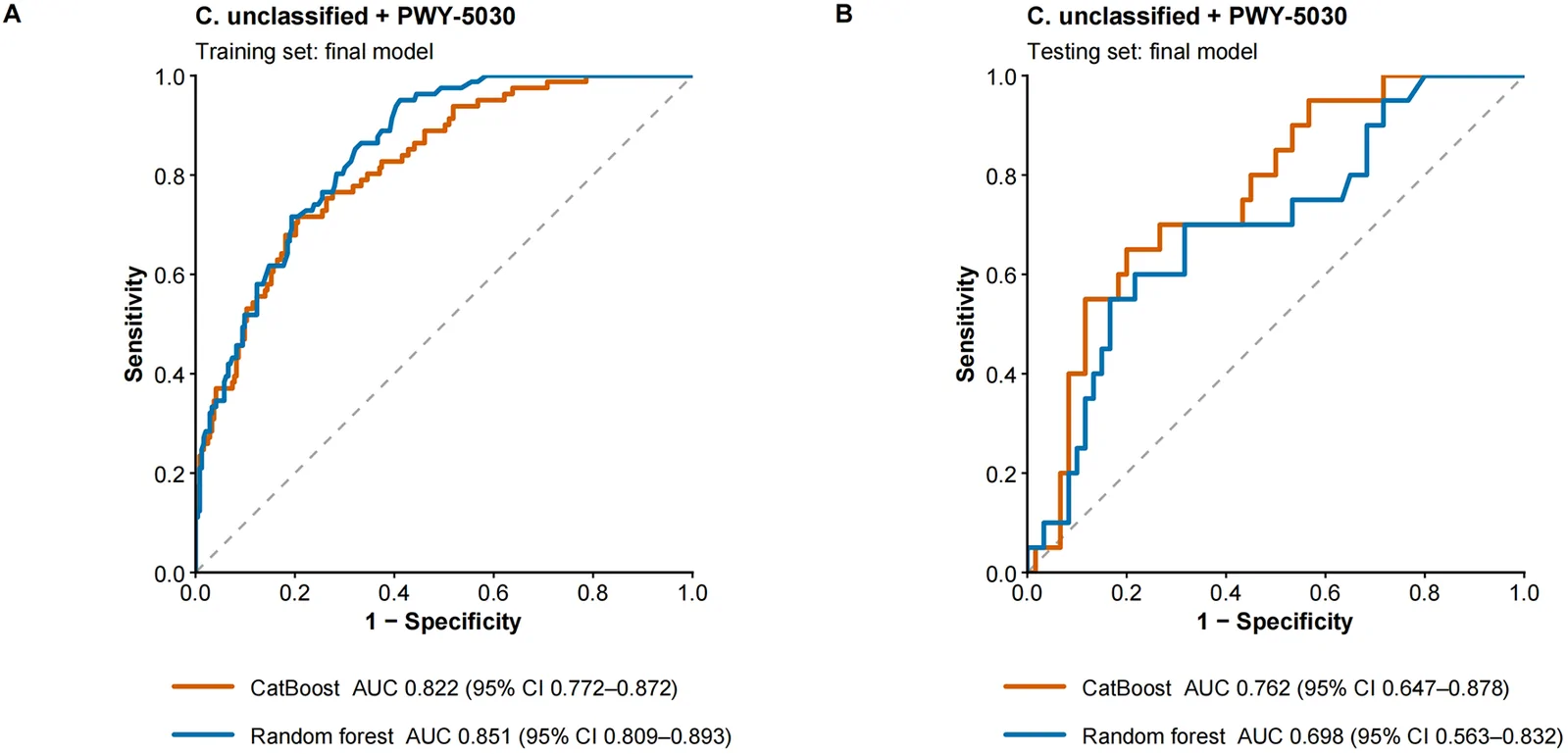
ROC curves of the final prediction models in the training and testing sets. **(A)** ROC curves of the CatBoost and RF models in the training set; **(B)** ROC curves of the CatBoost and RF models in the testing set.

To further assess the incremental predictive contribution of the gut microbial taxon and the associated metabolic pathway beyond traditional clinical indicators, three CatBoost models were constructed with progressively enriched feature sets: (1) the nine traditional indicators retained by LASSO regression; (2) these indicators plus *Coprobacillus unclassified*; and (3) these indicators plus both *Coprobacillus unclassified* and the metabolic pathway PWY-5030. In the independent test set, the model incorporating all 11 features (combination 3) consistently yielded the most favorable performance across evaluation metrics, including the highest area under the receiver operating characteristic curve, the most balanced sensitivity–specificity trade-off, and the largest F1 score, compared with combinations 1 and 2 (**Figure 6**). Notably, the addition of *Coprobacillus unclassified* alone (combination 2) produced only a marginal improvement over the traditional indicators, whereas further incorporating PWY-5030 (combination 3) resulted in a more pronounced enhancement, suggesting that the metabolic pathway may capture complementary NAFLD-related biological signals not fully represented by either the clinical indicators or the microbial taxon alone.

**Figure 6:**
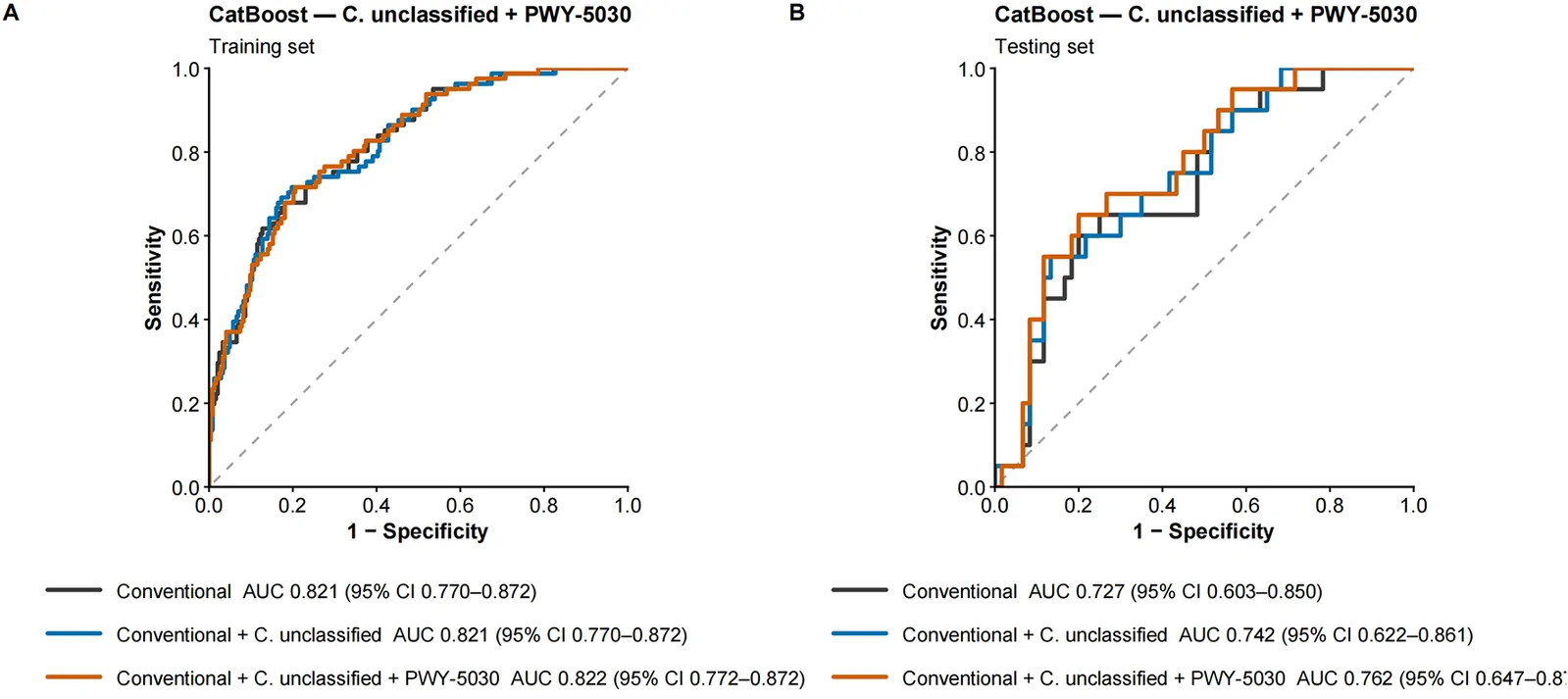
ROC curves of the CatBoost models with different feature combinations in the training and testing sets. **(A)** ROC curves of the CatBoost models based on conventional indicators only, conventional indicators + *Coprobacillus unclassified*, and conventional indicators + *Coprobacillus unclassified* + PWY-5030 in the training set; **(B)** the corresponding ROC curves in the testing set.

### SHAP analysis of the model

To quantify the contribution of each feature to the model output, the SHAP analysis was performed on the final CatBoost model. The feature importance plot, ranked by mean |SHAP| value, identified BMI, total protein, and WBC as the three most influential predictors, followed by drinking, waist circumference, total cholesterol, GGT, exercise, smoking, PWY-5030, and *Coprobacillus unclassified* (**Figure 7A**). The beeswarm plot simultaneously captured the relative importance of features and their relationship with the predicted outcome, with each dot representing a single subject, color-coded by the corresponding feature value and positioned along the x-axis according to its contribution to the predicted probability of NAFLD. Higher values of GGT, total cholesterol, drinking, smoking, PWY-5030, and *Coprobacillus unclassified* corresponded to positive SHAP values, indicating that elevated levels of these features were associated with an increased predicted probability of NAFLD. In contrast, higher exercise levels corresponded to negative SHAP values, suggesting a protective effect. Notably, lower values of BMI, total protein, WBC, and waist circumference were associated with positive SHAP values, whereas higher values were associated with negative SHAP values, implying non-monotonic or interaction-driven relationships between these clinical indicators and the predicted probability of NAFLD (**Figure 7B**).

**Figure 7:**
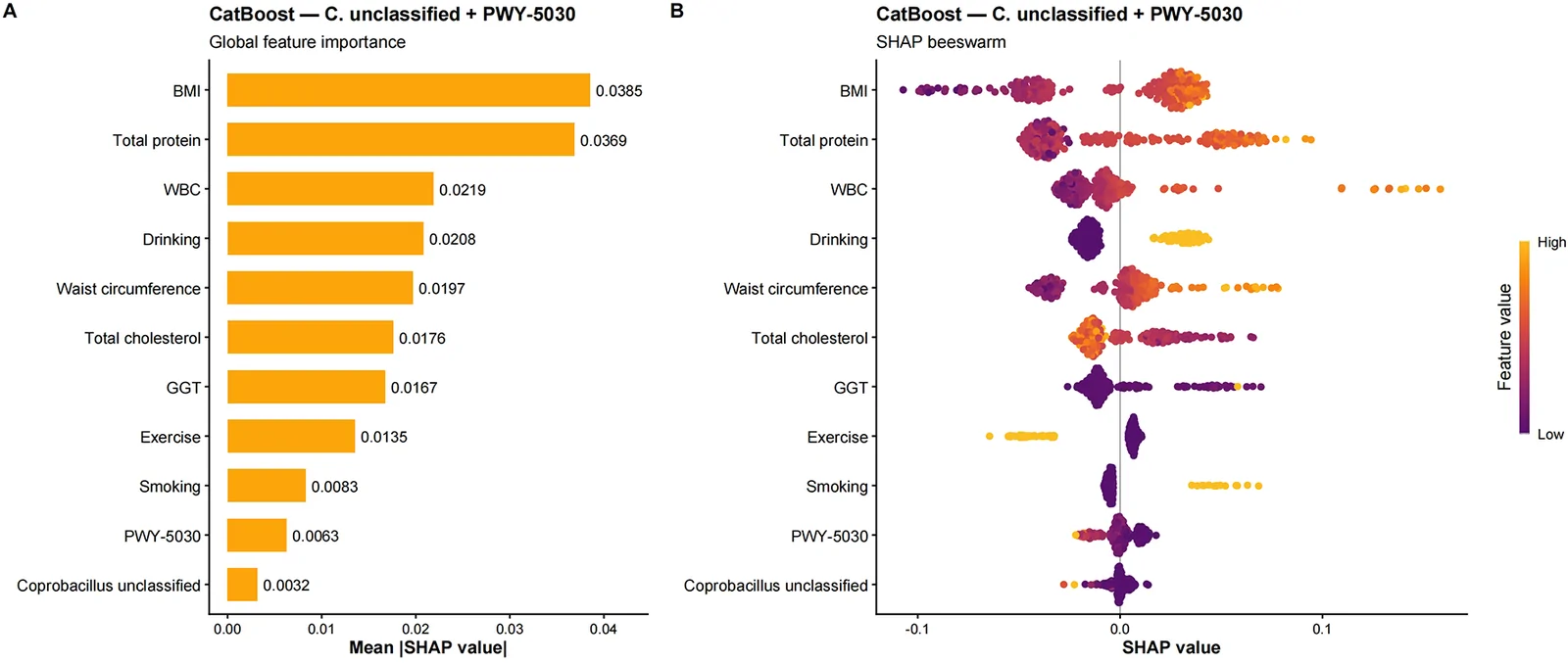
Model feature interpretation. **(A)** Feature importance ranking of the CatBoost model; **(B)** SHAP beeswarm plot of the CatBoost model.

## Discussion

In a brief period of time, the prevalence of NAFLD has significantly increased, especially in China (24). Among the most economically developed nations, China is expected to have the highest number of liver-related deaths by 2030 (25). An increasing amount of data suggests that the development of NAFLD may be linked to the gut microbiome (26). Numerous human investigations have shown that the gut microbiota of NAFLD patients differs from that of healthy people, as well as from that of people at different stages of the disease (27, 28). In the proposed concept of metabolic associated fatty liver disease (MAFLD), the gut microbiota is recognized as a key factor linked to the heterogeneous phenotypes of MAFLD (29). In research on NAFLD and MAFLD, multi-omics technologies that integrate patients’ clinical phenotypes, molecular phenomics, and gut microbial features can enhance the understanding of the complexity and heterogeneity of the disease.

In the results of this study, significant differences were observed in SBP and ALT between the NAFLD (-/+) group and the NAFLD (-/-) group (P<0.05), while no obvious differences were found in other liver function indicators including ALT, TP and ALB among all groups. Further investigation into the differential gut microbiota in the NAFLD (-/+) group revealed that the abundances of 36 microbial species (e.g., *Dorea formicigenerans*, *Bacteroides clarus*, *Alistipes finegoldii*, *Clostridium leptum*) were significantly increased in this group compared with the NAFLD (-/-) group, whereas the abundances of 8 species (e.g., *Bacteroides fragilis*, *Ruminococcus gnavus*, *Bacteroides dorei*, *Bacteroides caccae*) were significantly decreased. Based on these observations, we constructed a prediction model integrating gut microbiota features, microbial metabolic pathways, and clinical indicators.

The addition of *Coprobacillus unclassified* and PWY-5030 to the LASSO-selected clinical variables improved model performance, and this gain has a plausible biological basis. *Coprobacillus unclassified*, a member of the genus *Coprobacillus* (family Erysipelotrichaceae), was depleted in individuals who developed NAFLD. In a methionine- and choline-deficient mouse model of Non-alcoholic steatohepatitis (NASH), six weeks of butyrate supplementation attenuated hepatic injury, systemic inflammation, and gut-barrier dysfunction, and this protection was accompanied by enrichment of probiotic genera including *Coprobacillus (*30). Conversely, depletion of butyrate-associated taxa such as *Coprobacillus unclassified* may indicate an impaired short-chain-fatty-acid–mediated gut barrier, which facilitates endotoxin translocation and low-grade hepatic inflammation—processes central to NAFLD development (15, 30). In parallel, PWY-5030 encodes microbial L-histidine degradation. In three human cohorts totaling 651 individuals, Quesada-Vázquez et al. demonstrated that gut bacteria harboring histidine-utilization genes (principally Proteobacteria) catabolize host-derived histidine; lower circulating histidine was strongly and inversely associated with hepatic steatosis, and histidine supplementation reduced hepatic triglyceride accumulation and de no lipogenesis (DNL) in mouse, rat, and Drosophila models (31). A higher microbial capacity for histidine degradation may therefore reflect enhanced microbial consumption of this amino acid, limiting its systemic bioavailability and favoring hepatic lipid accumulation (31). Because neither the abundance of *Coprobacillus unclassified* nor microbial histidine catabolism is captured by routine clinical indicators, these two microbial features provide orthogonal, functionally interpretable information that plausibly underlies the observed gain in predictive performance.

Based on the findings regarding the specific alterations and functional analyses of the gut microbiota in NAFLD patients, the present study demonstrated that the gut microbiota in NAFLD may mediate glycolipid metabolism in patients and thereby induce NAFLD through multiple pathways: First, dysbiosis of NAFLD-specific gut microbiota (e.g., *Faecalibacterium prausnitzii*) may attenuate the effect of lactate and butyrate (major components of SCFAs) in improving insulin sensitivity. Meanwhile, the enrichment of NAFLD-specific gut microbiota (e.g., *Gegamonas rupellensis*) may lead to an increase in acetate and propionate (other SCFAs), which in turn exacerbates insulin resistance in patients. Second, specific alterations in the NAFLD-specific gut microbiota result in elevated intestinal LPS levels, thereby stimulating the production of proinflammatory cytokines, triggering a low-grade inflammatory response and reducing insulin sensitivity in patients. Third, the characteristic alterations in the gut microbiota of the NAFLD group affect host insulin sensitivity by generating aromatic amino acids (AAAs) through intestinal fermentation. Specifically, the depletion of NAFLD-specific gut microbiota may promote the fermentation of intestinal proteins to produce phenylalanine, which is further metabolized to phenol, ultimately impairing insulin sensitivity.

In clinical practice, the assessment of steatosis based on imaging techniques (e.g., MRS, CT or ultrasonography) has limitations such as high cost, radiation exposure and limited sensitivity. Therefore, numerous studies are striving to identify other reliable, cost-effective and non-invasive diagnostic methods. However, the application of currently widely used ML classification models, based on data of gut microbial composition and their metabolic pathways, is insufficient to achieve effective non-invasive identification and diagnosis of NAFLD.

This study aimed to explore the metagenomic data of NAFLD by applying a novel gradient boosting ensemble algorithm, namely CatBoost. A prediction model was constructed to predict NAFLD using gut microbiota profiles together with selected clinical indicators, and its predictive performance was evaluated by ROC analysis. The CatBoost model demonstrated promising predictive performance for NAFLD, with a test-set AUC of 0.762, suggesting its potential utility as a preliminary screening adjunct to existing clinical assessment for NAFLD risk stratification. In summary, this study preliminarily characterized the gut microbiome of individuals at risk of NAFLD and internally validated a risk prediction model integrating microbial, metabolic pathway, and clinical features. As an exploratory finding, these results support the feasibility of a non-invasive, microbiota-informed approach for early NAFLD risk assessment, although further external validation in larger and independent cohorts is required before clinical translation can be considered.

This study has several limitations. Liver biopsy, the gold standard for NAFLD diagnosis, was not adopted as the diagnostic criterion for cases and controls. Considering the large sample size, clinical feasibility and ethical constraints, it is impractical to perform liver biopsy on asymptomatic control participants. Although controls were selected using an age-matching strategy, sex distribution differed between the groups, which may have introduced residual confounding. Future studies with larger sample sizes and more balanced sex distributions are needed to validate our findings. Serum ferritin, an indicator closely associated with NAFLD, was not measured in this study. Incorporating this indicator and more biochemical markers may improve the predictive performance of the model, and the current prediction model needs further optimization for clinical application. In addition, this study cannot establish causal relationships between NAFLD and the identified taxonomic characteristics and metabolic profiles of the gut microbiota. Further longitudinal and mechanistic investigations are needed to substantiate these findings and elucidate the contribution of gut microbiota to NAFLD pathogenesis and progression. Moreover, this study lacks long-term follow-up gut microbiota data, making it impossible to assess the temporal dynamics of gut microbial composition. Meanwhile, the prediction model has not been validated by external independent cohorts, and its generalizability and clinical applicability require further validation.

## Conclusion

According to this study, the present findings indicate that gut microbiota alterations are closely associated with NAFLD and may represent candidate early markers of the disease. By applying machine learning to clinical, microbial and functional pathway data, this study illustrates the potential of non-invasive, multi-feature risk assessment. Future work will focus on clarifying the mechanisms linking the gut microbiota to NAFLD, thereby providing a firmer basis for microbiota-informed prevention and therapy. These findings contribute to the understanding of the microbiological mechanisms underlying NAFLD and offer a starting point for earlier detection, which may be further strengthened in independent populations.

## Data Availability

The datasets presented in this study can be found in online repositories. The names of the repository/repositories and accession number(s) can be found below: https://www.ebi.ac.uk/ena, PRJEB48022.
