# Supplementary material for: A nested case-control study on the relationship between gut microbiota and non-alcoholic fatty liver disease
Source: Front Endocrinol (Lausanne). 2026 Oct 2;17:1917815. doi: 10.3389/fendo.2026.1917815 (PMC13671956; doi:10.3389/fendo.2026.1917815)
Supplement: Supplementary file 1 [file Supplementaryfile1.docx]

Appendix Figures

**Fig. S1** Flow chart of study subject enrollment

**Fig. S2** Effects of baseline characteristics of study subjects on the gut microbiota

**Fig. S3** Comparison of α-diversity of the gut microbiota community between the NAFLD (-/+) and NAFLD (-/-) groups

**Fig. S4** Comparison of species-level β-diversity between the NAFLD (-/+) and NAFLD (-/-) groups based on PCoA analysis

**Fig. S5** Correlations between gut microbiota metabolic pathways and clinical indicators

**Fig. S6 Key predictive variable screening based on LASSO regression**

**Fig. S1** Flow chart of study subject enrollment

**Fig. S2** Effects of baseline characteristics of study subjects on the gut microbiota

**Fig S3** Comparison of α-diversity of the gut microbiota community between the NAFLD (-/+) and NAFLD (-/-) groups

**Fig. S4** Comparison of species-level β-diversity between the NAFLD (-/+) and NAFLD (-/-) groups based on PCoA analysis

**Fig. S5** Correlations between gut microbiota metabolic pathways and clinical indicators

**Fig. S6 Key predictive variable screening based on LASSO regression**. (A) Lasso regression feature selection plot, (B) Lasso coefficient convergence plot

Appendix Table

**Table S1** Relative abundances of gut microbial metabolic pathways with significant differences between the NAFLD (-/+) and NAFLD (-/-) groups

**Table S2** Functional classification of metabolic pathways

**Table S3** Metabolic Pathway Codes and Corresponding Names

**Table S1** Relative abundances of gut microbial metabolic pathways with significant differences between the NAFLD (-/+) and NAFLD (-/-) groups

| Metabolic pathways | NAFLD（-/+） | NAFLD（-/-） | *P* |
| --- | --- | --- | --- |
| SALVADEHYPOX-PWY | 1715.414±2111.575 | 1441.017±2477.567 | 0.319 |
| PWY-5367 | 1884.483±2015.396 | 1239.041±1521.759 | 0.004 |
| PWY-7117 | 1237.503±1748.125 | 1153.654±1506.776 | 0.642 |
| GLYCOCAT-PWY | 1522.439±1704.761 | 1256.139±1593.736 | 0.154 |
| PWY-5100 | 4923.069±2691.206 | 4517.030±2624.147 | 0.182 |
| GLUCUROCAT-PWY | 4577.768±2917.083 | 4562.373±2981.152 | 0.964 |
| PWY-6527 | 8822.205±6756.584 | 7335.770±4819.512 | 0.016 |
| GALACT-GLUCUROCAT-PWY | 4818.227±3016.674 | 4735.704±3005.390 | 0.811 |
| PWY-7242 | 4905.286±3275.790 | 4722.418±3169.226 | 0.619 |
| PWY-6507 | 4412.896±3119.875 | 4209.053±2974.266 | 0.556 |
| GALACTUROCAT-PWY | 5098.292±3376.137 | 4831.951±3199.214 | 0.475 |
| PWY66-422 | 10254.910±7419.998 | 8555.725±5282.078 | 0.035 |
| PWY-6317 | 10199.066±7419.300 | 8498.154±5274.374 | 0.035 |
| PWY-621 | 7624.129±4906.981 | 6420.535±3690.359 | 0.025 |
| PWY-6737 | 19643.293±9713.883 | 17415.083±8074.616 | 0.023 |
| TRNA-CHARGING-PWY | 3302.371±2831.743 | 2778.492±2095.384 | 0.090 |
| ARO-PWY | 17830.704±7880.876 | 16015.866±5760.804 | 0.035 |
| PWY0-1296 | 12385.309±7874.885 | 10527.199±5809.627 | 0.031 |
| DTDPRHAMSYN-PWY | 9343.222±4950.462 | 9394.086±5345.129 | 0.933 |
| PWY-5989 | 2604.725±2022.384 | 2011.610±1936.967 | 0.009 |
| CALVIN-PWY | 15606.446±9292.123 | 13243.477±7520.338 | 0.010 |
| GLYCOGENSYNTH-PWY | 4450.451±3142.818 | 4068.784±2990.899 | 0.274 |
| COBALSYN-PWY | 8265.623±7487.099 | 6307.535±5138.735 | 0.016 |
| GLCMANNANAUT-PWY | 7989.381±6524.188 | 6528.631±4601.138 | 0.039 |
| PWY-5030 | 709.768±700.003 | 866.880±937.776 | 0.123 |
| PWY-5345 | 1766.254±1641.278 | 1537.746±1728.621 | 0.245 |
| HSERMETANA-PWY | 2685.038±2001.351 | 2307.483±1523.154 | 0.048 |
| ASPASN-PWY | 8089.579±4167.565 | 7495.105±4068.852 | 0.207 |
| SER-GLYSYN-PWY | 6329.716±3618.562 | 6351.753±3849.968 | 0.960 |
| THRESYN-PWY | 9707.073±4823.782 | 8630.320±3974.714 | 0.026 |
| PWY-5101 | 3014.697±2780.688 | 3130.404±2673.114 | 0.709 |
| PWY-724 | 14217.484±6363.658 | 12547.381±4986.252 | 0.007 |
| PWY-3001 | 10790.038±5896.565 | 9505.135±4559.079 | 0.024 |
| BRANCHED-CHAIN-AA-SYN  -PWY | 13134.600±8281.180 | 11342.454±5862.792 | 0.046 |
| PWY-5103 | 11907.576±8030.344 | 10148.547±5652.677 | 0.043 |
| PWY-6122 | 14301.097±6201.736 | 12995.375±5032.296 | 0.034 |
| PWY-6277 | 14301.097±6201.736 | 12995.375±5032.296 | 0.034 |
| PWY-6121 | 14786.165±6111.697 | 13474.253±5133.595 | 0.035 |
| PWY-5177 | 2946.060±2186.882 | 2781.410±1993.467 | 0.484 |

**Table S2** Functional classification of metabolic pathways

| Functional Classification | NAFLD（-/+） | NAFLD（-/-） |
| --- | --- | --- |
| Nucleotide synthesis and degradation | PWY0-1296, PWY-6122, PWY-6277, PWY-6121, SALVADEHYPOX-PWY | PWY0-1297 |
| Carbohydrate utilization (glycolysis) | PWY-6527, PWY-6737, PWY-6507, PWY66-422, PWY-6317, PWY-621, PWY-7242, GLYCOCAT-PWY, GALACTUROCAT-PWY, GALACT-GLUCUROCAT-PWY,  GLUCUROCAT-PWY | GLYCOLYSIS |
| Lipid synthesis | PWY-5367 | PWY4FS-7, PWY4FS-8, PHOSLIPSYN |
| Energy conversion, nutrient metabolism and absorption | PWY-7117, PWY-5100, PWY-5177, ARO-PWY | PWY66-409, PWY-5484, ANAEROFRUCAT-PWY |
| Biomacromolecule synthesis | PWY-5989, PWY-5345, DTDPRHAMSYN-PWY, CAN-PWY, COSYN-PWY, GLYCOGENSYNTH-PWY | PWY-6700, PWY-1269, PWY-7539, PWY-6270, PWY-7560, PWY-5899, PWY-5897, PWY-5898, PWY-7228, PWY-6147, HEMESYN2-PWY |
| Biomacromolecule decomposition | PWY-5030, GLCMANNANAUT-PWY |  |
| Amino acid synthesis | PWY-5103, PWY-3001, PWY-724, PWY-5101, HSERMETANA-PWY, ASPASN-PWY, SER-GLYSYN-PWY, BRANCHED-CHAIN-AA-SYN-PWY, THRESYN-PWY | PWY-6628, PWY0-1061 |
| Amino acid activation | TRNA-CHARGING-PWY |  |
| Biomacromolecule decomposition | PWY-5030, GLCMANNANAUT-PWY |  |

**Table S3** Metabolic Pathway Codes and Corresponding Names

| Metabolic pathway | Group | Name |
| --- | --- | --- |
| PWY-6700 | NAFLD（-/-） | queuosine biosynthesis |
| PWY-7228 | NAFLD（-/-） | superpathway of guanosine nucleotides de novo biosynthesis I |
| PWY4FS-7 | NAFLD（-/-） | phosphatidylglycerol biosynthesis I (plastidic) |
| PWY4FS-8 | NAFLD（-/-） | phosphatidylglycerol biosynthesis II (non- plastidic) |
| PHOSLIPSYN | NAFLD（-/-） | Superpathway of phospholipid biosynthesis I (bacteria) |
| PWY-5484 | NAFLD（-/-） | glycolysis II (from fructose 6-phosphate) |
| GLYCOLYSIS | NAFLD（-/-） | glycolysis I (from glucose 6-phosphate) |
| ANAEROFRUCAT-PWY | NAFLD（-/-） | homolactic fermentation |
| PWY-6147 | NAFLD（-/-） | 6-hydroxymethyl-dihydropterin diphosphate biosynthesis I |
| HEMESYN2-PWY | NAFLD（-/-） | heme biosynthesis II (anaerobic) |
| PWY66-409 | NAFLD（-/-） | superpathway of purine nucleotide salvage |
| PWY0-1297 | NAFLD（-/-） | superpathway of purine deoxyrinucleosides degradation |
| PWY-1269 | NAFLD（-/-） | CMP-3-deoxy-D-manno-octulosonate biosynthesis I |
| PWY-7539 | NAFLD（-/-） | 6-hydroxymethyl-dihydropterin diphosphate biosynthesis III (Chlamydia) |
| PWY-6270 | NAFLD（-/-） | isoprene biosynthesis I |
| PWY-7560 | NAFLD（-/-） | methylerythritol phosphate pathway II |
| PWY-5899 | NAFLD（-/-） | superpathway of menaquinol-13 biosynthes- is |
| PWY-5898 | NAFLD（-/-） | superpathway of menaquinol-12 biosynthes- is |
| PWY-5897 | NAFLD（-/-） | superpathway of menaquinol-11 biosynthes- is |
| PWY-6628 | NAFLD（-/-） | superpathway of L-phenylalanine biosynthe- sis |
| PWY0-1061 | NAFLD（-/-） | superpathway of L-alanine biosynthesis |
| SALVADEHYPOX-PWY | NAFLD（-/+） | adenosine nucleotides degradation II |
| PWY-5367 | NAFLD（-/+） | petroselinate biosynthesis |
| PWY-7117 | NAFLD（-/+） | C4 photosynthetic carbon assimilation cycle, PEPCK type |
| GLYCOCAT-PWY | NAFLD（-/+） | Glycogen degradation I (bacterial) |
| PWY-5100 | NAFLD（-/+） | pyruvate fermentation to acetate and lactate II |
| GLUCUROCAT-PWY | NAFLD（-/+） | superpathway of &beta;-D-glucuronideand D-glucuronate degradation |
| PWY-6527 | NAFLD（-/+） | stachyose degradation |
| GALACT-GLUCUROCAT-PWY | NAFLD（-/+） | superpathway of hexuronide and hexuronate degradation |
| PWY-7242 | NAFLD（-/+） | D-fructuronate degradation |
| PWY-6507 | NAFLD（-/+） | 4-deoxy-L-threo-hex-4-enopyranuronatedegradation |
| GALACTUROCAT-PWY | NAFLD（-/+） | D-galacturonate degradation I |
| PWY66-422 | NAFLD（-/+） | D-galactose degradation V (Leloir pathway) |
| PWY-6317 | NAFLD（-/+） | galactose degradation I (Leloir pathway) |
| PWY-621 | NAFLD（-/+） | sucrose degradation III (sucrose invertase) |
| PWY-6737 | NAFLD（-/+） | starch degradation V |
| TRNA-CHARGING-PWY | NAFLD（-/+） | tRNAcharging |
| ARO-PWY | NAFLD（-/+） | chorismate biosynthesis I |
| PWY0-1296 | NAFLD（-/+） | purine ribonucleosides degradation |
| DTDPRHAMSYN-PWY | NAFLD（-/+） | dTDP-L-rhamnose biosynthesis I |
| PWY-5989 | NAFLD（-/+） | stearate biosynthesis II (bacteria and plants) |
| CALVIN-PWY | NAFLD（-/+） | Calvin-Benson-Bassham cycle |
| GLYCOGENSYNTH-PWY | NAFLD（-/+） | glycogen biosynthesis I (from ADP-D- Glucose) |
| COBALSYN-PWY | NAFLD（-/+） | adenosylcobalamin salvage from cobinami- de I |
| GLCMANNANAUT-PWY | NAFLD（-/+） | superpathway of N-acetylglucosamine, N- acetylmannosamine and N-acetylneuramin- ate degradation |
| PWY-5030 | NAFLD（-/+） | L-histidine degradation III |
| PWY-5177 | NAFLD（-/+） | glutaryl-CoA degradation |
| PWY-5345 | NAFLD（-/+） | superpathway of L-methionine biosynthesis (by sulfhydrylation) |
| HSERMETANA-PWY | NAFLD（-/+） | L-methionine biosynthesis III |
| ASPASN-PWY | NAFLD（-/+） | superpathway of L-aspartate and L-asparagi- ne biosynthesis |
| SER-GLYSYN-PWY | NAFLD（-/+） | superpathway of L-serine and glycinebiosy- nthesis I |
| THRESYN-PWY | NAFLD（-/+） | superpathway of L-threonine biosynthesis |
| PWY-5101 | NAFLD（-/+） | L-isoleucine biosynthesis II |
| PWY-724 | NAFLD（-/+） | superpathway of L-lysine, L-threonineand L-methionine biosynthesis II |
| PWY-3001 | NAFLD（-/+） | superpathway of L-isoleucine biosynthesis I |
| BRANCHED-CHAIN-AA-SYN-PWY | NAFLD（-/+） | superpathway of branched amino acid-biosy nthesis |
| PWY-5103 | NAFLD（-/+） | L-isoleucine biosynthesis III |
| PWY-6122 | NAFLD（-/+） | 5-aminoimidazole ribonucleotide biosynthe- sis II |
| PWY-6277 | NAFLD（-/+） | superpathway of 5-aminoimidazole ribonuc- leotide biosynthesis |
| PWY-6121 | NAFLD（-/+） | 5-aminoimidazole ribonucleotide biosynthesis I |
